# Integrated assessment of ecological land capability and land suitability for irrigated agriculture in Alborz Province Iran

**DOI:** 10.1038/s41598-026-38871-3

**Published:** 2026-02-06

**Authors:** Mohammad Saber Baghkhanipour, Romina Sayahnia, Naghmeh Mobarghaee Dinan, Afshin Danehkar

**Affiliations:** 1 Department of Environmental Planning and Design, Environmental Sciences Research Institute, Shahid Beheshti University, Tehran, Iran; 2 Department of Environmental Science, Faculty of Natural Resources, University of Tehran, Karaj, Iran

**Keywords:** Alborz province, Irrigated cereals, Land capability assessment, Land suitability evaluation, Sustainable agriculture, Spatial planning, Environmental impact, Agroecology, Ecological modelling

## Abstract

Increasing population density and improper land use have led to significant degradation of land resources, which is now a major environmental problem. A key challenge in sustainable agricultural planning is the separate application of land capability assessment (LCA) and land suitability assessment (LSE) methods in land spatial planning. The main objective of agricultural land suitability assessment is to assess the potential and constraints of land relative to the needs of target crops. This study aimed to develop a practical approach to integrate ecological LCA models with LSE models in Alborz province, Iran. Four cereal crops with a history of irrigated cultivation in the province were selected as target crops. The LCA model was integrated with the LSE model using nine factor-type and eight constraint-type criteria. The findings showed that approximately 7% of the province is suitable for irrigated cultivation of four crops, while the current irrigated agricultural area is approximately over 11%. A comparative analysis of two multi-criteria decision-making (MCDM) methods showed that calculating the programmable water amount and replacing monthly temperatures with continuity for target crops instead of the annual mean temperature by reflecting the potential agricultural capacity of the land and the initial crop composition increases the accuracy of the suitability measurement, which reduces the land identified as suitable for irrigated agriculture.

## Introduction

 Agricultural production in many emerging nations is insufficient to meet the needs of expanding population^1^. Globally, sustainable food production has become a critical challenge, prompting many countries to prioritize agricultural development policies within their national agendas^2^.The Sustainable Development Goals state that by 2030, nations must put in place resilient agricultural methods that boost productivity and output, support the maintenance of ecosystems, and have the ability to adapt to changes. These methods help strengthen climate resilience and gradually improve the quality of land and soil^3,4^.

Meeting the food needs of growing populations is complicated by multiple drivers, including climate change, urban and industrial expansion, and unsustainable land use. These pressures result in overexploitation of water and soil resources, land conversion, and loss of native species. Consequences include rural depopulation, migration to cities, soil erosion, reduced land productivity, groundwater depletion, dust storms, poverty, food insecurity, and economic instability.

Additionally, its effects include village depopulation and an increase in migration to cities, as well as land destruction, soil erosion, a reduction in the productive capacity of the land, depletion of underground water resources, the creation of dust centers, increased poverty among the populace, a threat to food security, and economic instability within society^5–9^.

Scholars emphasize sustainable resource use based on environmental protection, ecological understanding, and technologies adapted to demographic conditions^2^. Achieving sustainable agricultural production requires macro-level planning and policy evolution^10^.

Given limited resources, decision-makers must adopt advanced land assessment methods to identify suitable areas for food production^11^.Given the limited availability of additional agricultural areas, it will be necessary to increase crop yields through wise land use and management strategies in order to meet the nutritional demands of future populations^12^. Sustainable Land Management (SLM) integrates land, water, biodiversity, and environmental management to increase productivity while preserving ecological functions^13^. SLM is a knowledge-based approach that combines land, water, biodiversity, and environmental management to meet rising demand for food and fiber crops without hurting the ecosystem or the livelihoods of those involved^14^. SLM aims to integrate the key sectors of agriculture, and the environment with economic and social factors^15^.

In Iran, crop production patterns are not aligned with the ecological capacity of the land^16^. Land Capability Assessment (LCA) is essential for efficient agriculture, as sustainable development requires land use that matches ecological potential^17,18^. Multi-Criteria Decision-Making (MCDM) methods play a key role in identifying high-yield areas while minimizing environmental impacts^19–22^.

LCA evaluates current ecological conditions, including climate, water, soil, vegetation, and habitats, to determine appropriate land use. In contrast, LSE identifies land suitable for specific crops based on ecological requirements, facilitating a transition from general land use to crop-specific suitability^23^.

LSE refers to the appropriateness of a specific area for a particular type of land use, as well as the extent to which it meets the requirements of the land user. It is evident that LSE is influenced by various constraints associated with different land utilization practices. LSE is the first step in the development and enhancement of spatial land planning and in supporting sustainable agriculture^24^. Agricultural LSE offers crucial data for agricultural development and long-term planning for agriculturalproduction^25^ (AL-Taani et al., 2020). The fundamental goal of agricultural LSE is to forecast the potentials and constraints of land parcels in relation to the growth of certain crops^26^. One of the challenges in sustainable agriculture planning in the process of spatial land planning is the separate utilization of Land Capability Assessment (LCA) methods and Land Suitability Evaluation (LSE) methods. At present, MCDM on the development of agricultural crops in Iran are done only by relying on LSE methods, and no attention is paid to the ecological potential of land. Among others, we can refer to the research of Kamkar et al., 2021^27^, Servati et al., 2017^28^, Zahirnia and Matinfar 2019^29^, Navidi et al. 2022^30^ and Safari et al. 2017^31^. Combining these two strategies allows for the potential of attaining sustainable agriculture to provide food security, water security, and biological security in an integrated way. This may be done by coordinating the growth of agricultural operations with prudent land management. Therefore, main goal of this research is to provide a model of agricultural LCA (ecological capacity) that includes LSE.

Many decision-making issues in regional planning are multi-criteria issues. MCDM and evaluation analysis have become one of the most powerful methods in planning in the past few decades. Multi-criteria analysis includes methods that allow a range of criteria related to a topic to be ranked, scored, and weighted; then the desired options are prioritized and selected. Different MCDM models, and sometimes their integration, are heavily employed in locating and assessing the ecological capacity of diverse applications, including agricultural land use^32^. In environmental research and land management, multi-criteria and spatial multi-criteria MCDM models have been frequently used. These models are intended to achieve various goals, which, by studying the sources, can be summarized as follows: LCA, Locating and LSE, Land sensitivity and vulnerability assessment, Natural hazards risk assessment, Environmental monitoring and supervision and Environmental restoration^33^.Access to appropriate MCDM methods and emerging technologies can facilitate the pursuit of this goal^34–36^.

The main research questions were as follows:

- Is it possible to incorporate LSE criteria into an ecological LCA model?
- Which ecological criteria can be used to make the LCA model to incorporate LSE?

A spatial MCDM approach is LSE analysis^20,21^. The advantages of incorporating multi-criteria decision analysis techniques, particularly those that use spatial data, into LSE were recognized by several scholars^20,37–39^. Notable works carried out in this field include LSE for ecotourism^40^, land suitability analysis for agricultural crops^21,41^, LSE for urban development^42^, LSE for irrigated wheat cultivation^43^ and multi-criteria LSE analysis for livestock development^19^.

Previous studies (Tashayo et al., 2020^24^; Amini et al., 2019^44^; Naswati et al., 2019 ; Aymen et al., 2021^45^ ; Sobhani and Nasiri, 2016^46^; AbdelRahman, et al., 2019^47^; AbdelRahman, et al., 2019^48^) have applied diverse approaches to LSE, including GIS-AHP, fuzzy logic, and analytical network processes, across crops such as corn, rice, and wheat in Iran and other regions. These works highlight the importance of integrating spatial data and multi-criteria methods for sustainable agricultural planning.

## Materials and methods

### Study area

**Fig. 1:**
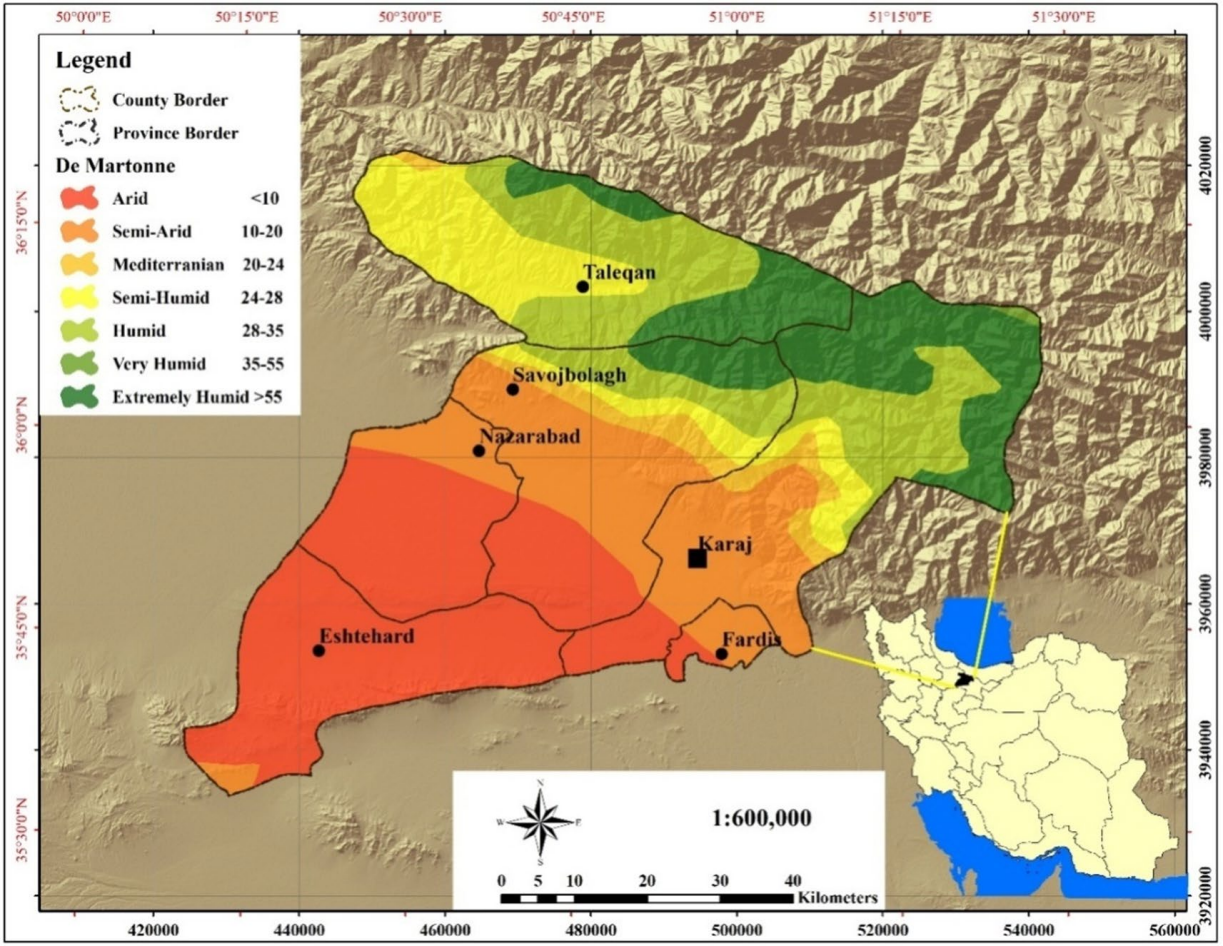
Location map of the study area. Map generated by the authors using QGIS 3.28 (https://qgis.org/download/), based on Table 2 data cited in the National Ecological Land Capability Assessment Report (https://civilica.com/doc/1716634).

The study area in this research is Alborz Province, Iran. This province covers an area of ​​5121.7 km^2^ located between 35°31′ and 36°12′ northern latitudes and 50°11′ and 51°29′ eastern longitudes. The cultivatable lands of the province lie within an elevation range of 900 and 1600 m above sea level^49^. Being located south of the Alborz Mountain range (Fig. 1), this province experiences two general climate type: cold and temperate in mountainous regions and warm and temperate in plains and desert regions. Alborz province, based on extended De Martonne climate classification, has the following climates types in the order of extent: arid, semi-arid, humid, very humid type 1, semi-humid, Mediterranean, and semi-humid type 2. Grade 2 and 3 rangelands cover around68% of the province area, followed by irrigated, rainfed, orchard, and fallow agricultural lands (19%), and developed areas including cities, towns, villages, industries, and infrastructure (7%), and bare terrain in the plains (5%)^50,51^. Less than 0.5% of Alborz Province is covered with scattered mountain forests and river valley groves, and about the same proportion is watered by both natural and artificial rivers and waterbodies^49^.

### Research methodology

**Fig. 2:**
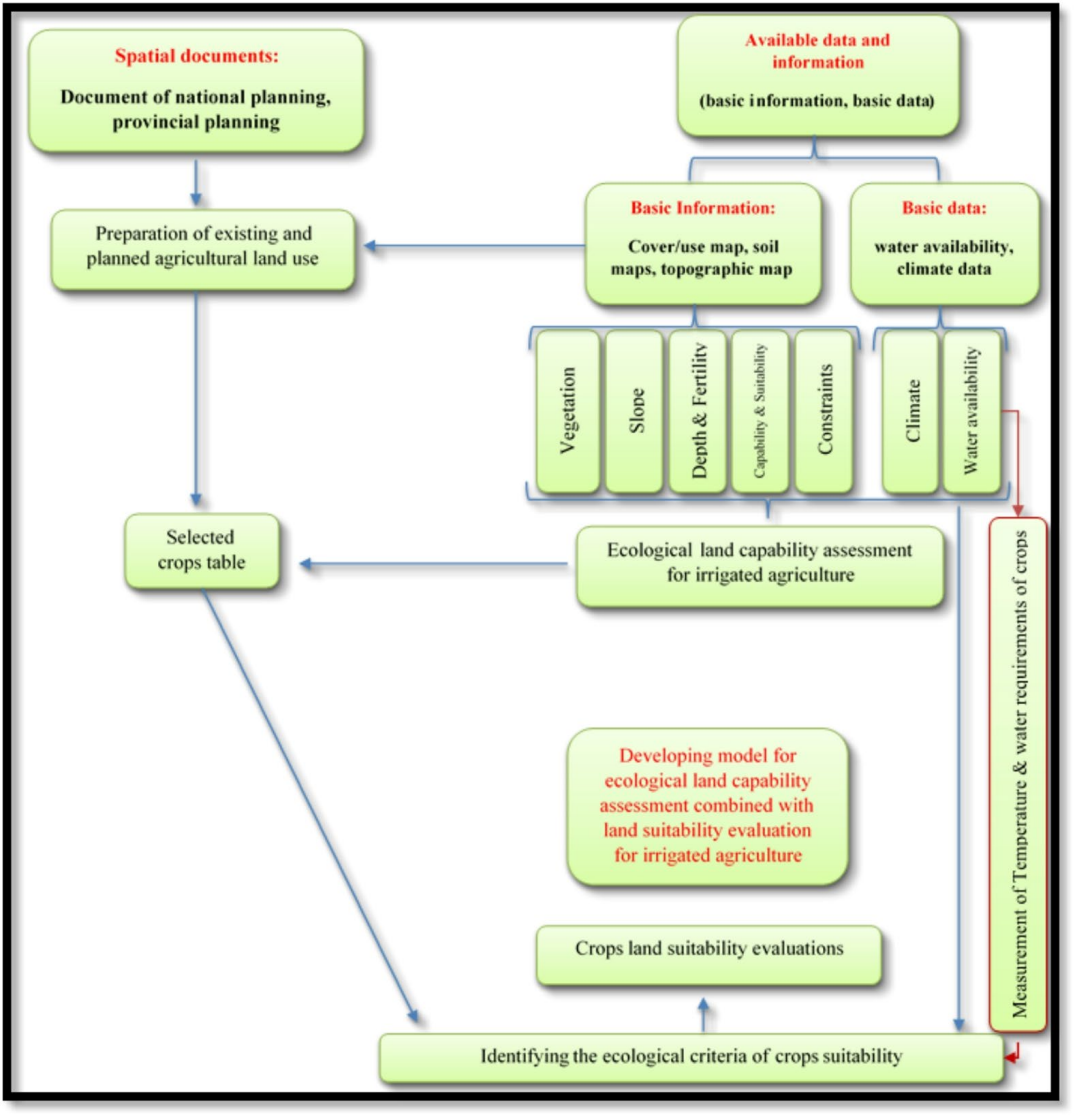
Research conceptual model.

This study was interdisciplinary, descriptive-exploratory research with applied dimensions. It falls in the category of applied research in terms of purpose, and classifies as documentary-survey research because of using documentary. LCA methods for spatial land planning purposes rely on ecological LCA models. In the context of agriculture, these models determine which areas have a capacity for agricultural development (irrigated or rainfed), subject to land constraints. In Iran, conventional LCA models can only identify agriculturally-capable territories, but not the types of crops that would be suitable based on ecological characteristics. To resolve this issue, it is necessary to develop a new LCA model with the ability to determine the range of suitable crops for a given agricultural development project. It was assumed that agricultural development will take place via increased production of 4 crops that are already being cultivated in the study area: wheat, barley, maize, and sorghum. The LCA model was integrated with the LSE model for these crops to enhance the practicality of the LCA model for the target crops. Figure 2 illustrates the conceptual model of the research.

This study was conducted in three distinct stages, the methodology of which is detailed in the following subsections.

### LCA method

In ecological LCA for agricultural purposes, it is common to use a number of capability criteria (so-called factors) plus a number of constraints in linear LCA models^52,53^.Competent ecological characteristics for irrigated agriculture are among the spatial parameters that might influence capacity evaluation. In 1993, Makhdoom created the first ecological LCA-MCDM model for Iran. This model operates based on four main ecological criteria, including water availability (in the range of 3000 to 10000 cubic meters per hectare per year), terrain roughness (slopes of less than 12%), soil characteristics (texture, depth, fertility, drainage), and climate. This model was updated and expanded by Danehkar et al. based on the country’s existing basic plans for the years 2017 to 2020. In addition to separating irrigated and rainfed agriculture, these researchers modified the MCDM model for irrigated farming so that it considers competence and constraint variables in relation to five ecological criteria, namely climate (average temperatures higher than 5 °C), terrain roughness (altitudes of less than 3000 m above sea level, slopes of less than 10%), soil characteristics (suitability for farming, soil orders, depth, fertility, drainage, and salinity), water availability in the basin (over 100 cubic meters per hectare per year) and natural vegetation (palatable rangelands and all forest habitats as constraint parameters) with improved precision.

With one adjustment, the soil order map of the irrigated agricultural LCA model was swapped out for the “resource and land assessment classes” (land unit classes) map in this study’s ecological LCA. The ecological LCA model of this study is provided in Table 1. In this model, agricultural sustainability is pursued with enough attention to environmental hazards (soil erosion and flooding), as well as those areas where agricultural development is not possible (culturally or ecologically significant areas, and areas within compatible land uses) so that assessment results can be used for conflict-free MCDM.

**Table 1:** Framework of the irrigated agriculture ecological LCA model for Alborz province.

Row	Criteria	Competency range of capability indicators	Constraint index range
1	Slope class	Up to 10%	More than 10%
2	Elevation sea level class	Up to 1600 m	More than 1600 m
3	Average annual temperature	More than 5 °C	Less than 5 °C
4	Soil suitability class for agriculture	I, IIIA, IIIS, IIIST, IIIT, IIIW + VA, IIS, IIST, IIT	IVS, IVT
5	Land unit	2.1, 3.7, 3.21,4.2, 6.11, 7.9, 8.1, 8.4, 9.1, 9.8	1.1, 1.2, 1.4, 1.6, 2.3, 2.7, 2.9, 4.18, 4.25, 4.26
6	Soil depth class	Low to very high	Very low depth
7	Soil fertility class	Medium to good	Weak and very weak
8	Soil drainage class	Weak to good	–
9	Soil salinity class	–	High to very high
10	Forest cover	–	All forest habitats
11	Rangeland cover	Rangelands with the production of less than 200 kg/ha and coverage of less than 25%	Rangelands with palatability species, produce more than 200 kg and cover more than 25%
12	Water availability in the basin	More than 100 m^3^/ha/year	Less than 100 m^3^/ha/year
Constraint due to natural hazards			
13	Soil erosion class	–	High to severe
14	Flooding class	–	Severe
Constraint due to incompatible land uses			
15	Culturally significant areas	–	Areas of ancient and historical value (buffer 500 m)
16	Ecologically significant areas	–	National Park, national natural monument (not available in the province), wetlands, natural lakes, permanent rivers (buffer 150 m)
17	Incompatible land use	–	Existing cities and industrial towns, lakes behind the dam (buffer 150 m)

The relevant national sources and the Alborz province’s spatial land planning database were used to gather the necessary information related to the aforementioned criteria. Table 2 contains a list of these data. The information layers corresponding to the criteria were prepared using GIS equipment (QGIS software) and classified based on competency ranges (Table 3). Considering the nature of criteria (compensating and non-compensating), union/intersection patterns of competency ranges for each criterion were integrated in the format of a linear model (Eq. 1) under the framework used in Danehkar’s national ecological LCA study^53^ to reach a linear LCA model for irrigated farming. This linear model is of the non-weighted type, since it is mandatory to include the criteria in MCDM. Then, the linear model layers were combined to generate an ecological LCA zoning map for irrigated farming.

Equation (1) linear model of irrigated farming LCA for Alborz province

1 $$\begin{aligned} {\mathrm{IA}} & ={\mathrm{SL1}}+{\mathrm{EL1}}+\left( {{\mathrm{TE2}},{\mathrm{TE3}},{\mathrm{TE4}}} \right)+\left( {{\mathrm{SS1}},{\mathrm{SS2}}} \right)+{\mathrm{LU1}}+\left( {{\mathrm{SD1}},{\mathrm{SD2}},{\mathrm{SD3}}} \right)+\left( {{\mathrm{SR1}},{\mathrm{SR2}},{\mathrm{SR3}},{\mathrm{SR4}}} \right) \\ & \quad +\left( {{\mathrm{SF1}},{\mathrm{SF2}},{\mathrm{SF3}}} \right)+\left( {{\mathrm{AW2}},{\mathrm{AW3}},{\mathrm{AW4}}} \right) - \left( {{\mathrm{SA3}},{\mathrm{FT1}},{\mathrm{RO1}},{\mathrm{EH2}},{\mathrm{FH2}},{\mathrm{PO1}},{\mathrm{PO2}},{\mathrm{PO3}}} \right) \\ \end{aligned}$$

In this equation, IA is the suitable zone for irrigated farming, SL denotes the slope class, EL denotes the elevation class, TE denotes the average temperature class, SS denotes the soil suitability class, LU denotes the land unit class, SD denotes the soil depth class, SF denotes the soil fertility class, SR denotes the soil drainage class, AW denotes the water availability class, SA denotes soil salinity class, FT denotes the forest cover class, RO denotes the non-developable rangeland class, EH denotes soil erosion class, FH denotes the flood susceptibility class, and PO denotes the class of culturally, historically, ecologically sensitive areas and areas with incompatible land uses.

**Table 2:** Information layers used in the LCA and LSE of the study area.

Row	Information layer	Scale	References
1	Elevation classes	1:250,000	Topographic map of Iran National Cartographic Center
2	Slope classes	1:250,000	Topographic map of Iran National Cartographic Center
3	Isothermal classes	1:250,000	Data of Iran Meteorological Organization - Topographic map of Iran National Cartographic Center
4	Soil suitability classes	1:100,000	LCA map prepared by Soil and Water Research Institute of Iran
5	Soil depth classes	1:100,000	LCA map prepared by Soil and Water Research Institute of Iran
6	Soil fertility classes	1:100,000	LCA map prepared by Soil and Water Research Institute of Iran
7	Soil drainage classes	1:100,000	LCA map prepared by Soil and Water Research Institute of Iran
8	Soil salinity classes	1:250,000	LCA map prepared by Soil and Water Research Institute of Iran
9	Land unit classes	1:100,000	LCA map prepared by Soil and Water Research Institute of Iran
10	Classes of water availability for agricultural	1:250,000	Comprehensive plan of water resources management - Iranian Ministry of Energy
11	Forest type classes	1:250,000	Vegetation map - Natural Resources and Watershed Management Organization of Iran
12	Forest dense classes	1:250,000	Vegetation map - Natural Resources and Watershed Management Organization of Iran
13	Rangeland type classes	1:250,000	Vegetation map - Natural Resources and Watershed Management Organization of Iran
14	Rangeland palatability classes	1:250,000	Vegetation map - Natural Resources and Watershed Management Organization of Iran
15	Rangeland dense classes	1:250,000	Vegetation map - Natural Resources and Watershed Management Organization of Iran
16	Classes of protected areas	1:250,000	Iran’s Department of Environment
17	Soil erosion classes	1:250,000	LC map prepared by Soil and Water Research Institute of Iran
18	Flooding classes	1:100,000	Geological map of Geological Survey and Mineral Exploration of Iran
19	Areas of ancient and historical value	1:25,000	Topographic map of Iran National Cartographic Center
20	Water resources (rivers and water bodies)	1:100,000	Geological map of Geological Survey and Mineral Exploration of Iran
21	Land use	1:50,000	Land cover and land use map of Alborz province - Provincial spatial land planning document

**Table 3:** Irrigated farming LCA and LSE classes for Alborz province.

Criteria	Class 1	Class 2	Class 3	Class 4
% Slope				
Range	0–10	> 10		
Code	SL1	SL2		
Elevation class				
Range	1600>	> 1600		
Code	EL1	EL2		
Average annual air temperature (ºC)				
Range	5>	5–10	10–15	15–20
Code	TE1	TE2	TE3	TE4
Average monthly air temperature (ºC)				
Range	2–8	8–14	14–30	30–40
Code	TM1	TM2	TM3	TM4
Growth period (days)				
Range	90–100	100–120	120–130	130–160
Code	GC1	GC2	GC3	GC4
Soil suitability				
Range	I	IIIA, IIIS, IIIST, IIIT, IIIW + VA, IIS, IIST, IIT	IVS, IVT	
Code	SS1	SS2	SS3	
Land unit				
Range	2.1, 3.7, 4.2, 8.1, 8.4, 9.1	1.1, 1.2, 1.4, 1.6, 2.3, 2.7, 3.1,4.1		
Code	LU1	LU2		
Soil depth (cm)				
Range	High (75< )	Relatively high (50–75)	Relatively low to low (50 − 25)	Very low (25> )
Code	SD1	SD2	SD3	SD4
Soil fertility				
Range	Good	Relatively good	Medium	Weak
Code	SF1	SF2	SF3	SF4
Soil drainage				
Range	Good	Medium	Imperfect	Weak
Code	SR1	SR2	SR3	SR4
Soil salinity				
Range	Very low	Low	Low to medium	High to very high
Code	SA0	SA1	SA2	SA3
Forest cover				
Range	Without cover	Has cover		
Code	FT0	FT1		
Rangeland cover				
Range	Production less than 200 kg and coverage less than 25%	Palatability species, produce more than 200 kg and cover more than 25%		
Code	RO1	RO2		
Water availability (m^3^/hectare/year)				
Range	100>	100–500	500–1000	1000<
Code	AW1	AW2	AW3	AW4
Soil erosion				
Range	Very low to medium	Relatively high to very high		
Code	EH1	EH2		
Flooding				
Range	Relatively mild	Medium	Severe	
Code	FH0	FH1	FH2	
Non-developable				
Range	Historical-cultural	Ecologically significant	Incompatible	
Code	PO1	PO2	PO3	

### LSE method

LSE models rely on the analyses conducted for a series of target crops. Such LSE models can be described as an environment friendly approach to land use management for agricultural development. In this approach, target crops are assessed based on their ecological requirements and the range of geographic factors in the environment under study. Four different varieties of cereals that have historically been grown under irrigation in the region of Alborz were the study’s target crops. Since it could be challenging to identify the right environmental criteria for agricultural productivity, researchers proposed different criteria with varying components and scopes for this purpose. However, these criteria were determined using the book “Land Evaluation”^54^ as the main reference and other related articles serving as supplementary references.

Accordingly, the information available in the NETWAT^55^ software database of the study area, and data from scientific articles was used to extract criteria for target crop and their reported ranges of values^55–58^. It should be noted that these factors were eliminated from the table of LSE criteria (Table 4) to prevent interference, since NETWAT determines the net irrigation water demand based on effective precipitation (in millimeters) and evapotranspiration(in millimeters).

Furthermore, in the mentioned table, temperature range and minimum water availability were assumed to correspond to a yield of over 40%. The long-term meteorological data of Alborz province from local synoptic stations and their linear gradients were used to generate temperature zone maps based on the needs of each crop and the time required to achieve the minimum planned production. Isotherm maps for all months of the year were also obtained. Table 5 shows the average monthly temperature in 16 meteorological stations of Alborz province in the period from 1956 to 2019.

The data needed for LSE was applied in accordance with Table 2. Based on linear correlations between the ranges of appropriate values of the criterion for each crop, LSE mapping for the target crops was carried out using the minimal range of parameters provided in Table 4 for yields above 40%. The LSE conducted in this study only covered those parts of the province that had either agricultural land use or no designated land use at all.

**Table 4:** Range of irrigated agriculture suitability criteria for the target cereals.

Criteria	% production	Wheat	Barley	Maize	Sorghum
Growth period (days)	–	100–130	120–160	90–130	90–130
Water availability for agriculture (m^3^/hectare)	50	500	500	1200	1300
	75	700	700	3100	3100
	100	1500<	1500<	4000<	4000<
% Slope	40>	10<	10<	10<	10<
	40–60	8–10	10<	8–10	8–10
	60–85	4–8	8–10	4–8	4–8
	85–95	2–4	2–8	2–4	2–4
	95–100	0–2	0–2	0–2	0–2
Average monthly air temperature (ºC)	40>	8> 30<	2> 42<	12> 40<	12> 32<
	40–60	8–10 26–30	2–4 36–42	14–16 34–40	14–18
	60–85	10–12 24–26	4–6 30–36	16–18 32–34	18–22
	85–95	12–14 20–24	6–8 24–30	18–22 26–32	22–24 26–32
	95–100	14–20	8–24	22–26	24–26
Soil depth (cm)	40>	10>	10>	10>	10>
	40–60	10–20	10–20	20–50	10–20
	60–85	20–50	20–50	50–75	20–50
	85–95	50–90	50–90	75–100	50–90
	95–100	90<	90<	100<	90<
Soil drainage	40>	Very weak	Very weak	Very weak	Very weak
	40–60	Weak	Weak	Weak	Weak
	60–85	Imperfect	Imperfect	Imperfect	Imperfect
	85–95	Medium	Medium	Medium	Medium
	95–100	Good	Good	Good	Good
Soil salinity	40>	Very weak	Very weak	Very weak	Very weak
	40–60	Weak	Weak	Weak	Weak
	60–85	Medium	Medium	Medium	Medium
	85–95	Good	Good	Good	Good
	95–100	Excellent	Excellent	Excellent	Excellent

**Table 5:** Average monthly and annual temperatures of meteorological stations in the study area.

Elevation (m)	Station name	Parameter	October	November	December	January	February	March	April	May	June	July	August	September	Annual
1417	Deh Somee	Average	14.94	7.82	2.77	0.79	2.57	7.02	12.21	16.63	21.45	23.88	23.31	19.80	12.77
1220	Valadabad	Average	13.99	7.38	1.38	− 2.64	− 0.05	6.90	13.14	17.80	22.18	25.23	24.55	20.51	12.53
1193	Karimabad	Average	16.21	10.09	4.24	0.24	0.74	5.35	11.34	16.54	22.30	26.05	26.01	22.45	13.46
1886	Asara	Average	11.93	5.11	− 1.42	− 3.72	− 2.01	2.92	9.62	14.71	20.14	23.89	22.90	18.42	10.21
2170	Nesa	Average	10.42	4.33	− 1.88	− 4.37	− 3.19	1.37	7.76	12.68	17.35	20.52	19.89	16.15	8.42
2530	Gagere	Average	10.13	4.96	− 0.60	− 4.74	− 6.60	− 2.97	2.59	9.23	13.59	17.84	18.32	15.36	6.43
2173	Shahrestang	Average	10.51	4.25	− 1.65	− 4.79	− 2.81	1.76	7.39	12.47	17.46	21.22	20.96	16.62	8.62
1322	Karaj	Average	17.11	9.91	4.56	1.81	4.12	8.67	14.53	19.21	24.58	27.10	26.69	22.74	15.09
1311	Haidarabad	Average	15.59	8.08	4.45	1.61	2.16	7.46	12.90	17.06	22.16	24.21	23.90	21.56	13.43
1807	Karajmicroclimatology	Average	15.37	9.13	2.07	− 0.72	1.07	7.07	13.55	18.31	23.64	26.27	24.45	21.77	13.58
1726	Karaj Dam	Average	14.57	8.28	3.43	1.12	2.63	6.58	12.22	17.07	22.18	24.83	24.01	20.50	13.12
1652	Solghan	Average	14.27	7.83	3.21	1.53	2.59	5.35	12.43	17.45	23.42	26.10	24.97	21.04	13.35
1953	Jostan	Average	14.49	7.97	2.76	0.66	− 0.69	3.04	8.03	13.08	18.31	21.95	22.65	19.88	11.01
2155	Darvan	Average	12.95	7.45	0.70	− 2.65	− 3.03	− 0.02	6.08	11.55	16.95	22.25	22.70	19.65	9.55
1885	Zidasht	Average	14.14	7.43	1.61	− 1.59	− 1.68	1.85	7.74	12.69	18.19	22.09	22.68	19.76	10.41
1807	Glink	Average	11.85	3.42	− 2.14	− 5.03	− 3.99	− 0.83	5.46	10.42	15.61	20.27	20.55	16.96	7.71

### Method of LSE-based ecological LCA

**Fig. 3:**
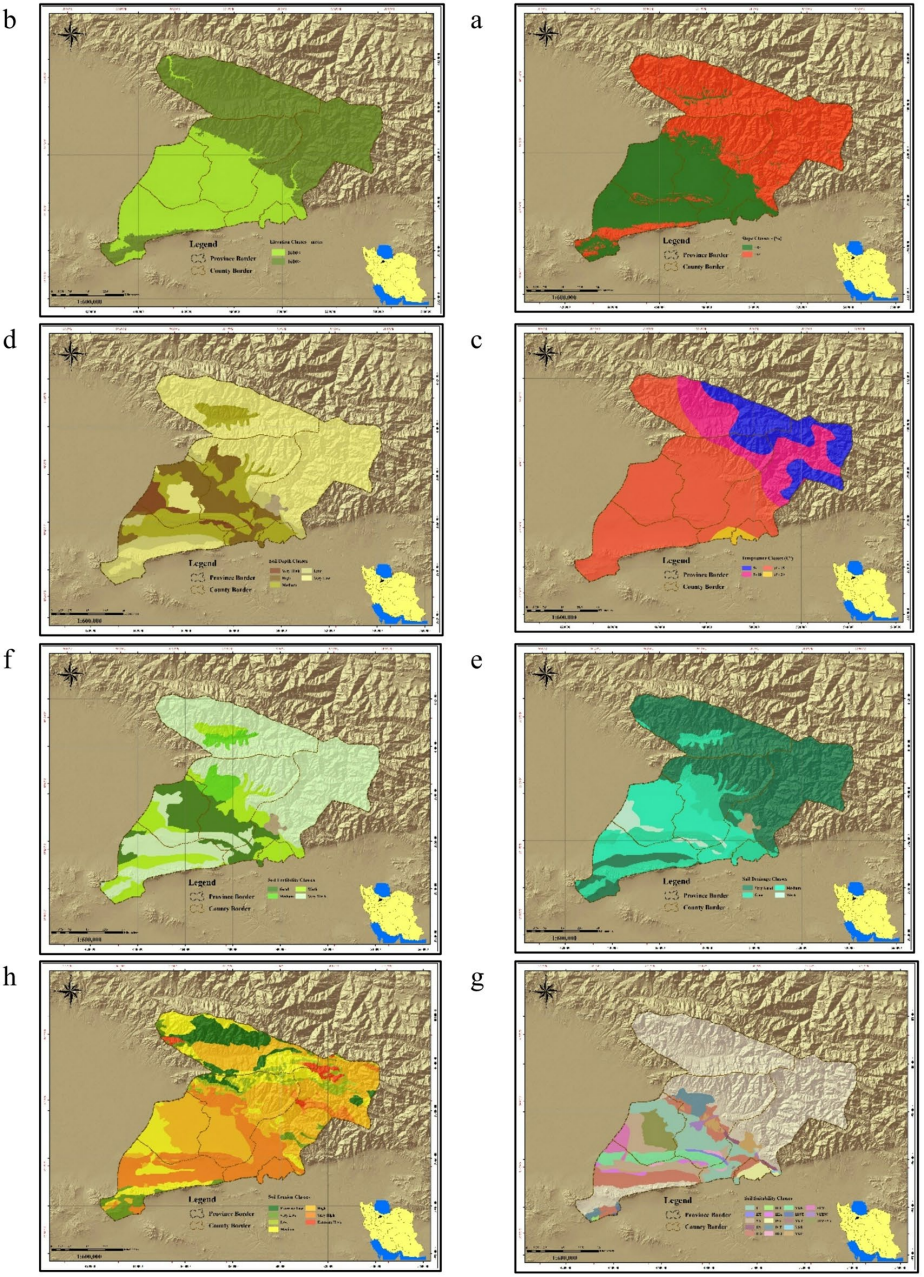

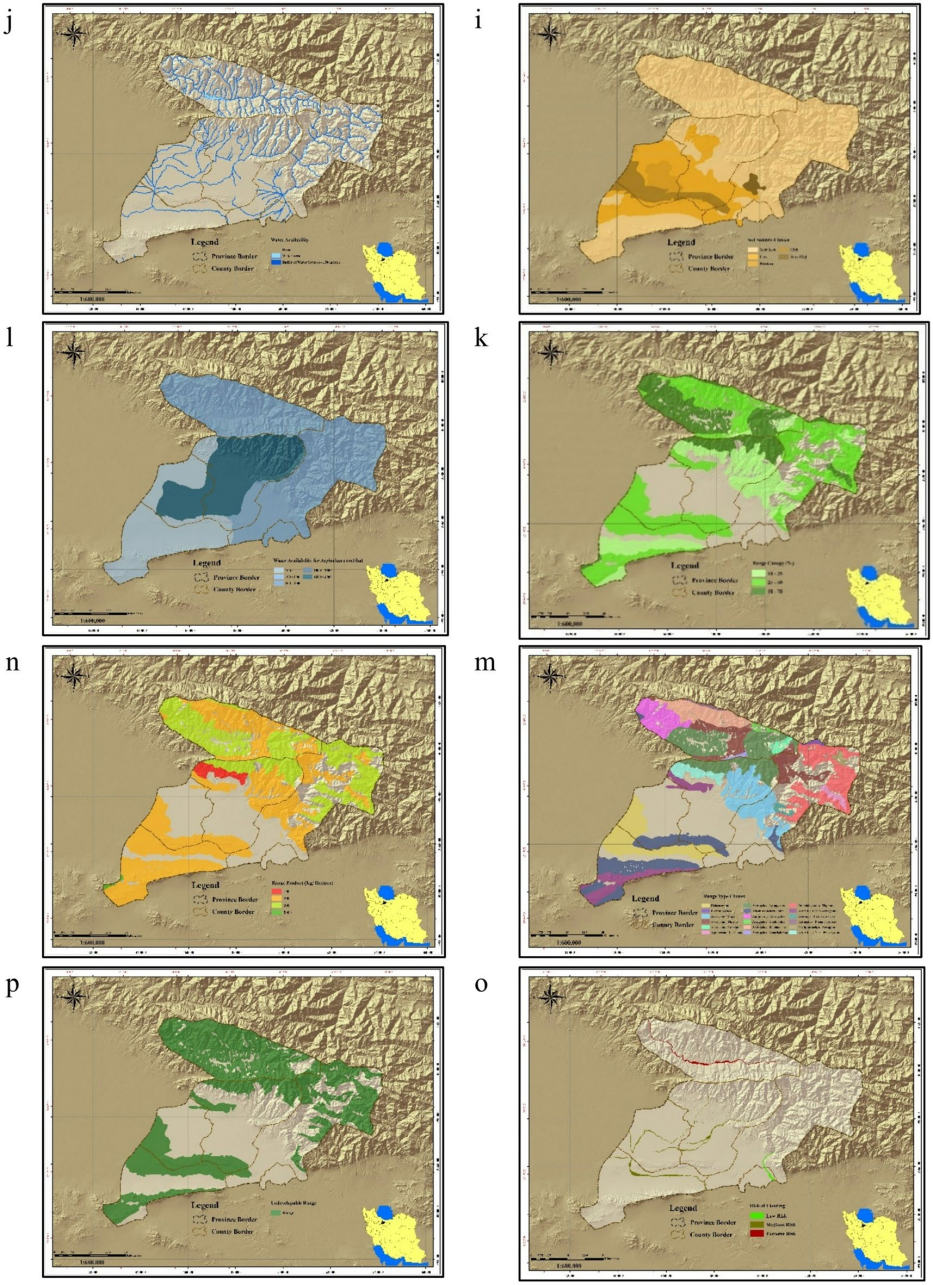

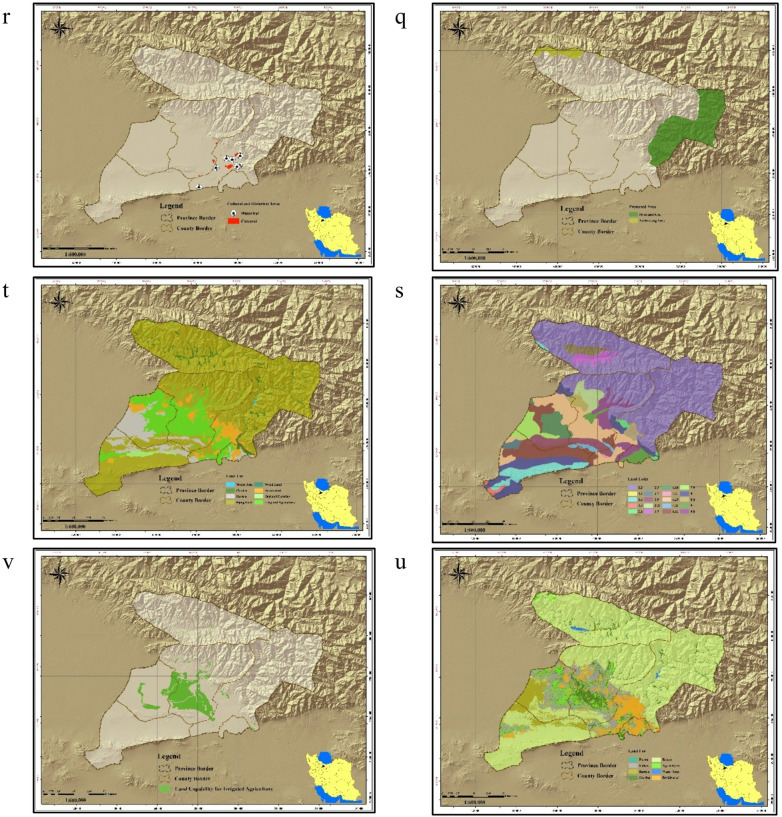
Thematic maps used in ecological land capability assessment; (**a**) slope classes; (**b**) elevation classes; (**c**) temperature classes; (**d**) soil depth; (**e**) soil drainage; (**f**) soil fertility; (**g**) soil suitability; (**h**) soil erosion; (**i**) soil salinity; (**j**) water availability; (**k**) rangeland canopy; (**l**) water availability for agriculture; (**m**) rangeland types; (**n**) rangeland productivity; (**o**) flood risk; (**p**) undevelopable rangelands; (**q**) protected areas; (**r**) cultural and historical areas; (**s**) land units; (**t**) land use map (national planning); (**u**) land use map (provincial planning); (**v**) ecological land capability map for irrigated agriculture. Map generated by the authors using QGIS 3.28 (https://qgis.org/download/), based on Table 2 data cited in the National Ecological Land Capability Assessment Report (https://civilica.com/doc/1716634).

The integration of information maps in GIS produces a map of suitability zones for the target crops. Following the approach used in previous models and the one applied in Danehkar’s national ecological LCA^53^, the LCA model was developed by the aggregating of the value range of the criteria from the LSE models for the selected crops. Next, this model was modified to incorporate LSE into LCA, enabling it to identify both the potential for product development and the agricultural activity group (irrigated). This leads to more practical results from spatial land zoning and relates land plans to agricultural calendar, as well as spatial patterns, thereby increasing the added value gained from agricultural land. This innovation in applying ecological LCA models can serve as a platform for improved spatial land planning. After preparing the LCA model based on the suitability of agricultural crops, the ecological LCA was repeated to generate another map. This map can be compared with the ecological LCA maps (Fig. 3, u, v) presented in master (provincial or national) land spatial planning documents to achieve more accurate LSE maps.

## Results

### LCA results

LCA of the study area for the development of irrigated agriculture was performed using the descriptive model (Table 1) and linear relationship (Eq. 1) obtained for the area. The LCA analysis produced criteria maps for nine factors and eight constraints (Fig. 3a–v). Precipitation in the study area ranged from < 200 to 1000 mm, indicating that crops requiring higher rainfall are unsuitable unless supplemented by groundwater. Water availability varied between < 100 and 2000 m³/ha/year, with most of the area falling in the 500–1000 m³/ha/year class (Fig. 3l). Soil drainage ranged from good to poor, while fertility was predominantly very poor (Fig. 3f). Average monthly temperatures ranged from < 5 °C to 20 °C, with the 10–15 °C range covering the largest area. Slopes < 10%—most suitable for irrigated farming—were the least frequent class (Fig. 3a). Integration of these criteria in QGIS (Eq. 1) identified lands with ecological capability for irrigated farming (Fig. 3v).

### LSE results

**Fig. 4:**
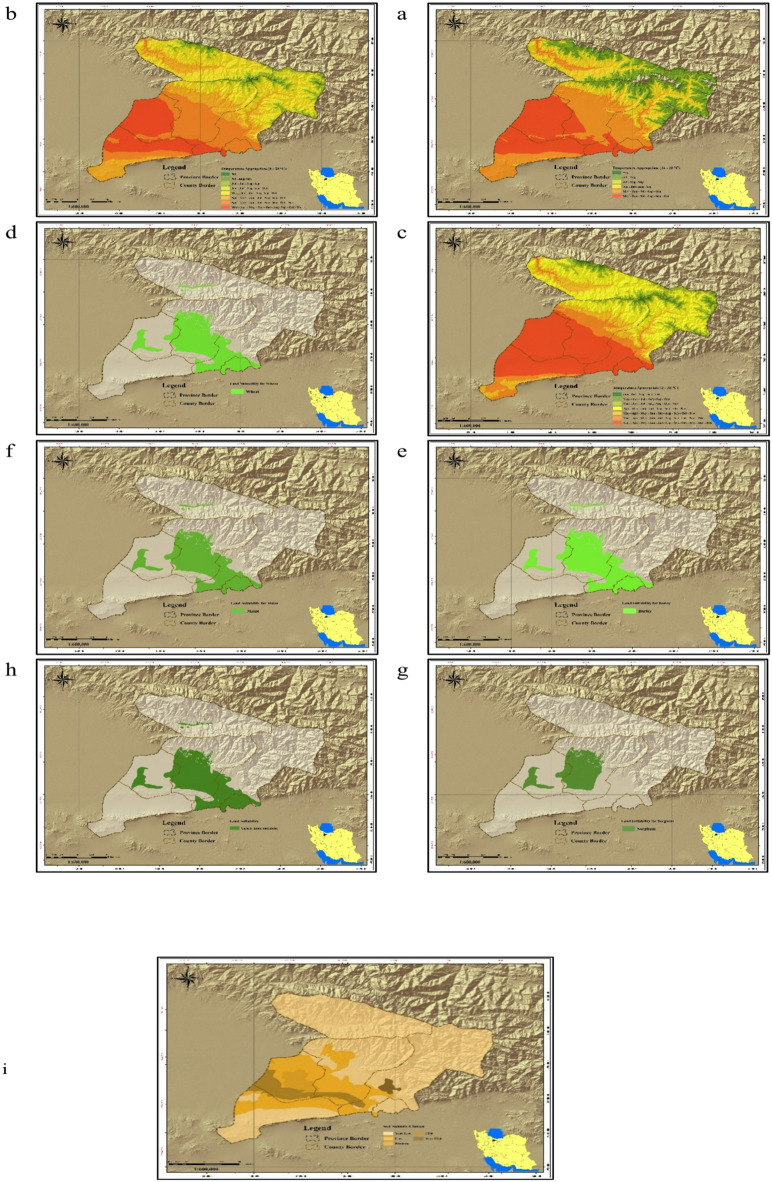
Thematic maps used in ecological land capability assessment; (**a**) temperature appropriate map (14–28 °C); (**b**) temperature appropriate map (8–28 °C); (**c**) temperature appropriate map (2–28 °C); (**d**) wheat land suitability evaluation (LSE) map; (**e**) barley LSE map; (**f**) maize LSE map; (**g**) sorghum LSE map; (**h**) cereals LSE map; (**i**) ecological land capability–suitability map. Map generated by the authors using QGIS 3.28 (https://qgis.org/download/), based on Table 2 data cited in the National Ecological Land Capability Assessment Report (https://civilica.com/doc/1716634).

LSE was conducted for four cereal crops using climate, soil, water, and topography criteria. Barley tolerated the widest temperature range (> 40 °C), while wheat and barley could grow below 10 °C. Most crops required medium to excellent soil fertility (≥ 1% organic carbon). Suitability maps (Fig. 3a–u) were generated for each criterion. Linear relationships between ecological parameters were derived for each crop (Eqs. 2–5), enabling crop-specific suitability mapping. A critical determinant was temperature continuity: maize and sorghum required 90–130 consecutive days of suitable temperatures, wheat 100–130 days, and barley 120–160 days (Table 6). The optimal range for all crops was 14–30 °C, supporting > 95% yield over five months (Fig. 4a). Other ranges (30–32 °C, 8–40 °C, 2–42 °C) supported progressively lower yields (Fig. 4b–c). Final suitability maps for wheat, barley, maize, and sorghum are shown in Fig. 4d–h.

Equation (2) Linear relationship of combinations of irrigated wheat farming LSE criteria for Alborz province

2 $$\begin{aligned} {\mathrm{IAW}} & ={\mathrm{SL1}}+\left( {{\mathrm{TM2}},{\mathrm{TM3}}} \right)+\left( {{\mathrm{SD1}},{\mathrm{SD2}},{\mathrm{SD3}},{\mathrm{SD4}}} \right)+\left( {{\mathrm{SR1}},{\mathrm{SR2}},{\mathrm{SR3}}} \right) \\ & \quad +\left( {{\mathrm{SF1}},{\mathrm{SF2}},{\mathrm{SF3}},{\mathrm{SF4}}} \right)+\left( {{\mathrm{AW2}},{\mathrm{AW3}},{\text{ AW4}}} \right) \\ \end{aligned}$$

Equation (3) Linear relationship of combinations of irrigated barley farming LSEcriteria for Alborz province

3 $$\begin{aligned} {\mathrm{IAB}} & ={\mathrm{SL1}}+\left( {{\mathrm{TM1}},{\mathrm{TM2}},{\mathrm{TM3}},{\mathrm{TM4}}} \right)+\left( {{\mathrm{SD1}},{\mathrm{SD2}},{\mathrm{SD3}},{\mathrm{SD4}}} \right)+\left( {{\mathrm{SR1}},{\mathrm{SR2}},{\mathrm{SR3}}} \right) \\ & \quad +\left( {{\mathrm{SF1}},{\mathrm{SF2}},{\mathrm{SF3}},{\mathrm{SF4}}} \right)+{\text{ }}\left( {{\mathrm{AW2}},{\mathrm{AW3}},{\mathrm{AW4}}} \right) \\ \end{aligned}$$

Equation (4) Linear relationship of combinations of irrigated maize farming LSE criteria for Alborz province

4 $${\mathrm{IAM}}={\mathrm{SL1}}+\left( {{\mathrm{TM2}},{\mathrm{TM3}},{\mathrm{TM4}}} \right)+\left( {{\mathrm{SD1}},{\mathrm{SD2}},{\mathrm{SD3}},{\mathrm{SD4}}} \right)+\left( {{\mathrm{SR1}},{\mathrm{SR2}},{\mathrm{SR3}}} \right)+\left( {{\mathrm{SF1}},{\mathrm{SF2}},{\mathrm{SF3}},{\mathrm{SF4}}} \right)+\left( {{\mathrm{AW4}}} \right)$$

Equation (5) Linear relationship of combinations of irrigated sorghum farming LSE criteria for Alborz province

5 $${\mathrm{IAS}}={\mathrm{SL1}}+\left( {{\mathrm{TM2}},{\mathrm{TM3}},{\mathrm{TM4}}} \right)+\left( {{\mathrm{SD1}},{\mathrm{SD2}},{\mathrm{SD3}},{\mathrm{SD4}}} \right)+\left( {{\mathrm{SR1}},{\mathrm{SR2}},{\mathrm{SR3}}} \right)+\left( {{\mathrm{SF1}},{\mathrm{SF2}},{\mathrm{SF3}},{\mathrm{SF4}}} \right)+\left( {{\mathrm{AW4}}} \right)$$

### Results of ecological LCA based on LSE

Integration of LCA and LSE through MCDM enhanced model performance by incorporating temperature continuity. Comparative analysis of two MCDM methods showed that adding temperature ranges allowed outputs to reflect both land capacity and crop composition. Temperature–humidity requirements for the four crops are summarized in Table 7. Using these criteria, ecological LCA maps were generated (Fig. 4i). Replacing average annual temperature with monthly temperature continuity (Table 8) improved accuracy, effectively transforming the LCA into a capability–suitability assessment model. This integrated approach provides more reliable identification of suitable lands for irrigated cereal cultivation in Alborz Province.

**Table 6:** Temperature requirements of the target crops based on percent yield.

Product	Wheat	Maize	Sorghum	Barley
Growth period	100–130 days	90–130 days	90–130 days	120–160 days

Average monthly temperature	% Production			
42>	40>	40>	40>	40>
40–42	40>	40>	40>	40–60
38–40	40>	40–60	40>	40–60
36–38	40>	40–60	40>	40–60
34–36	40>	40–60	40>	60–85
32–34	40>	60–85	40>	60–85
30–32	40>	85–95	85–95	60–85
28–30	40–60	85–95	85–95	85–95
26–28	40–60	85–95	85–95	85–95
24–26	60–85	95–100	95–100	85–95
22–24	85–95	95–100	85–95	95–100
20–22	85–95	85–95	60–85	95–100
18–20	95–100	85–95	60–85	95–100
16–18	95–100	60–85	40–60	95–100
14–16	95–100	40–60	40–60	95–100
12–14	85–95	40>	40>	95–100
10–12	60–85	40>	40>	95–100
8–10	40–60	40>	40>	95–100
6–8	40>	40>	40>	85–95
4–6	40>	40>	40>	60–85
2–4	40>	40>	40>	40–60

**Table 7:** Temperature-humidity requirements of the target crops in the study area.

Average monthly temperatures °C	Growth period	Water availability (m^3^/hectare/year)			
		100–500	500–1000	1000–1500	1500–2000
14–30	3 months	Wheat	Wheat, Maize	Wheat, Maize, Sorghum	Wheat, Maize, Sorghum
30–32		–	Wheat, Maize	Maize, Sorghum	Maize, Sorghum
8–40		Wheat	Wheat, Maize	Wheat, Maize, Sorghum	Wheat, Maize, Sorghum
2–40		–	–	–	–
14–30	4 months	Wheat, Barley	Wheat, Barley, Maize	Wheat, Barley, Maize, Sorghum	Wheat, Barley, Maize, Sorghum
30–32		Barley	Barley, Maize	Barley, Maize, Sorghum	Barley, Maize, Sorghum
8–40		Wheat, Barley	Wheat, Barley, Maize	Wheat, Barley, Maize, Sorghum	Wheat, Barley, Maize, Sorghum
2–42		Barley	Barley	Barley	Barley
14–30	5 months	Barley	Barley	Barley	Barley
30–32		Barley	Barley	Barley	Barley
8–40		Barley	Barley	Barley	Barley
2–42		Barley	Barley	Barley	Barley

**Table 8:** New temperature criterion and its appropriate range for upgrading the ecological LCA model for the target crops.

Constraint	Capability	Criterion
Average monthly temperatures below 2 °C and over 42 °C	Continuous average monthly temperatures of 2–42 °C over a period of 1–5 months, subdivided into four ranges: 14–30 °C, 30–32 °C, 8–40 °C, and 2–42 °C	Suitable air temperature

## Discussion

Anthropogenic depletion of natural resources has raised concerns about ecosystem health and global food security. Sustainable Land Management (SLM) is widely recognized as a key strategy to prevent land degradation and ensure long-term agricultural productivity. In this study, SLM is considered within the framework of Land Capability Assessment (LCA) and Land Suitability Evaluation (LSE), which together provide a basis for aligning agricultural practices with ecological conditions.

The anthropogenic depletion and destruction of natural resources, which has caused major concerns regarding ecosystem health and global food security, is rooted in humans’ misconceptions about nature and the consequent misuse of natural resources. It is vital to assess the food security status of both urban and rural communities and to identify the factors influencing this status, given the importance of food security in achieving comprehensive development and fostering a healthy, active population^59^. Therefore, SLM can be viewed as a protective measure against land degradation, aimed at preventing the destruction of land resources. In this context, one may ask: what is the exact definition of land degradation? The Global Environment Facility defines land degradation as any decline in the natural potential of land that adversely affects an ecosystem’s integrity by reducing its ecological productivity, natural bioavailability, and recovery capacity. According to the World Bank, Sustainable Land Management (SLM) is essential for meeting the needs of the world’s growing population. This international institution warns that poor land management can lead to land degradation, resulting in a significant reduction in a nation’s productive and service outputs^14^. Therefore, SLM can be viewed as a protective measure against land degradation, aimed at preventing the destruction of land resources. In this context, one may ask: what is the exact definition of land degradation? The Global Environment Facility defines land degradation as any decline in the natural potential of land that adversely affects an ecosystem’s integrity by reducing its ecological productivity, natural bioavailability and recovery capacity^60,61^. One of the most important aspects of Sustainable Land Management (SLM) is the alignment of agricultural activities with environmental conditions^2^. Therefore, Land Capability Assessments (LCAs) are crucial for efficient agriculture, as optimal land use requires an accurate assessment of ecological resources12. Land Suitability Evaluation (LSE) is the first step in the development and enhancement of spatial land planning, and in supporting sustainable agriculture19. In the context of agriculture, this process is referred to as LSE, and its primary goal is to forecast the potentials and constraints of individual land parcels in relation to the production of specific crops21. This study makes three main contributions: (1) updating and applying an ecological LCA model for irrigated agriculture in Alborz Province, (2) integrating constraint-type criteria into LCA, which are often neglected, and (3) combining LCA with crop-specific LSE to improve accuracy by incorporating temperature continuity. However, the original word-based model of Makhdoom (1993) requires updating and modification a matter that was duly noted by some users but overlooked by others. It is important to note that in all cases involving the use of LCA models, decisions were made based on criteria related to the land’s ecological resources. However, the fundamental ecological SLM model, utilized in the nation’s national spatial land planning document44 and customized for Alborz Province, was employed to conduct the LCA for irrigated agriculture in this research. All ecological LCA models operate based on the assumption that having optimal ecological conditions is a necessary requirement for realizing the maximum land potential. The basic principle of LCA in these models is to evaluate the natural capability of the environment without any remediation or artificial adjustment. In other words, these models measure the inherent ecological potential of the environment43.Weighting methods are generally not well-suited for ecological assessments and tend to be ineffective when applied exclusively to ecological data (i.e., physical and biological resources)51. Therefore, it is reasonable to avoid weighting ecological criteria in LCA, which is why this approach was deliberately excluded from this study. The LCA model used for irrigated agriculture in Alborz Province included nine capability criteria and eight constraints. Several other studies including Chen et al. (2021)^62^, Altani et al. (2020)^25^, Di Feudis et al. (2020)^63^, Nguyen et al. (2020)^64^, Mazahreh et al. (2019)^26^, Atalay (2016)^65^, Akinsi et al. (2013)^21^, Safaripour and Naseri (2020)^66^, Bidadi et al. (2015)^17^, and Pourkhabbaz et al. (2015)^18^ have applied these criteria in LCA. However, very few studies have incorporated constraint-type criteria in this MCDM process.

This neglect stems from the fact that the nature of LCA tends to direct the researcher’s focus toward capability factors, while drawing attention away from constraint factors. However, it is important to remember that careful consideration of restriction factors is essential for managing capability components and establishing precise spatial boundaries. In reality, in situations where progress may be severely limited, capability elements alone are insufficient. Application of the irrigated agriculture LCA model revealed that only 6% of Alborz Province is suitable for irrigated agriculture. This contrasts sharply with the provincial spatial land planning document^49^, which identified 19% as suitable for agriculture and 11% for irrigated agriculture. The difference highlights the importance of incorporating ecological constraints into LCA, as conventional planning may overestimate land suitability. The irrigated agriculture LCA model used in this study does not account for irrigated orchards, particularly those located on sloping lands. This indicates that such agricultural systems require a dedicated and specialized model tailored to their unique ecological and spatial characteristics. In the context of agriculture, Land Suitability Evaluation (LSE) must be conducted for specific target crops. In this study, the target crops consisted of four cereal types, for which four main LSE criteria climate, soil, water availability and topography were applied, along with nine sub-criteria: monthly average temperature, soil suitability, land unit, soil drainage, soil depth, soil fertility, available water, and slope. According to the study’s findings, 7% of the province is suitable for producing these four major cereal crops, with a considerable portion of that area being ideal for cultivating common cereals with strong ecological adaptability, such as maize, wheat, and barley.

It is important to note that careful attention to the ecological requirements of each specific crop not only enables more accurate delineation of suitability zones, but also expands the spatial scope of LSE, especially when temperature ranges overlap across both monthly and seasonal periods (e.g., 2–42 °C).

These findings are consistent with previous studies that applied GIS-AHP, fuzzy logic, and other MCDM approaches to crop suitability analysis in Iran and abroad(Tashayo et al. (2020)^24^, Amini et al. (2019)^44^, Neswati et al. (2019)^12^, Aymenet al. (2021)^45^, Sobhani and Nasiri, (2020)^46^. The present study extends this work by explicitly integrating LCA and LSE, and by incorporating constraint-type criteria and temperature continuity into the assessment.

## Conclusion

This study aimed to develop a practical solution for integrating ecological LCA models with crop-centered LSE models in the agricultural sector. Since both models rely on ecological criteria, it was hypothesized that they could be combined through modification of decision components and refinement of the appropriate range of environmental factors. In addition to confirming this hypothesis, the results demonstrated that a capability-based LSE model can be effectively constructed by replacing average annual temperature with average monthly temperature, incorporating temperature continuity (based on the growth period of target crops), and improving the accuracy of minimum water availability mapping using grade 4 basins (609 basins across Iran).

The model’s performance was validated through a case study in Alborz Province, involving four cereal crops. It successfully refined the results of the irrigated agriculture LCA model while producing overlapping outcomes with the LSE model for agricultural development in the region. Given that approximately 200 agricultural crops are currently cultivated in Iran, the development of this MCDM model will significantly contribute to spatial land planning, facilitating the creation of knowledge-based agricultural development plans aligned with LCA principles. The results of such assessments can be used to generate agricultural species distribution maps that align with ecological LCA, thereby supporting efforts to achieve and maintain food security. Also, given that in land planning studies, the needs of agricultural plant species and crop suitability are not considered in assessing the ecological potential of irrigated agricultural lands, the results of this finding can improve this decision-making system.

## Suggestions

- Integration of Economic and Social Dimensions as complementary research, it is recommended to incorporate economic and social components into environmental suitability studies. Factors such as market access, agricultural policies, and farmers’ technology adoption significantly influence land use decisions. Including these dimensions will help develop a more comprehensive decision support tool for policymakers and enhance the practical applicability of ecological models.
- Enhancement of Soil Data Accuracy Field-level investigations require more accurate soil profile data and detailed soil descriptions. Additionally, attention to parameters such as micro-nutrient availability and soil composition variability would significantly improve the precision of land suitability assessments.
- Inclusion of Crop-Specific Needs in Land Planning Current land planning studies often overlook the specific ecological requirements of agricultural plant species and product suitability when assessing the potential of irrigated agricultural lands. Addressing this gap can enhance the decision-making framework. This recommendation is directed to the Planning and Budget Organization of the Iranian Government, as a key authority in national land use policy.
- Expansion to Other Agricultural Land Types This study focused exclusively on irrigated agricultural lands. Future research should extend the scope to include rainfed, orchard, and fallow lands, thereby enabling a more holistic evaluation of agricultural potential across diverse land types.

## Data Availability

The data used and analyzed during this study are available from the corresponding author upon reasonable request.
